# Reducing Socioeconomic Disparities in Comprehensive Smoke-Free Rules among Households with Children: A Pilot Intervention Implemented through a National Cancer Program

**DOI:** 10.3390/ijerph17186787

**Published:** 2020-09-17

**Authors:** Michael J. Parks, Michelle C. Kegler, John H. Kingsbury, Iris W. Borowsky

**Affiliations:** 1 Institute for Translational Research in Children’s Mental Health, University of Minnesota, Minneapolis, MN 55415, USA; 2 Department of Behavioral Sciences & Health Education, Rollins School of Public Health of Emory University, Emory University, Atlanta, GA 30322, USA; mkegler@emory.edu; 3 Office of Statewide Health Improvement Initiative, Minnesota Department of Health, St. Paul, MN 55101, USA; john.kingsbury@state.mn.us; 4 Division of General Pediatrics and Adolescent Health, Department of Pediatrics, Medical School, University of Minnesota, Minneapolis, MN 55414, USA; borow004@umn.edu

**Keywords:** smoke-free rules, tobacco-related disparities, socioeconomic disadvantage, secondhand smoke exposure, child and adolescent health

## Abstract

Most households with a smoker do not implement comprehensive smoke-free rules (smoke-free homes *and* cars), and secondhand smoke (SHS) exposure remains prevalent among children and low-socioeconomic status (SES) populations. This pilot project aimed to assess implementation feasibility and impact of an intervention designed to increase smoke-free rules among socioeconomically disadvantaged households with children. The pilot was implemented through Minnesota’s National Breast and Cervical Cancer Early Detection Program (NBCCEDP). NBCCEDPs provide cancer prevention services to low-income individuals experiencing health disparities. We successfully utilized and adapted the Smoke-Free Homes Program (SFHP) to address comprehensive smoke-free rules among households with children. We used two recruitment methods: (a) direct mail (DM) and (b) opportunistic referral (OR) by patient navigators in the NBCCEDP call center. We used descriptive statistics to assess implementation outcomes and hierarchical logistic regression models (HLM) to assess change in smoke-free rules and SHS exposure over the study period. There was no comparison group, and HLM was used to examine within-person change. A total of 64 participants were recruited. Results showed 83% of participants were recruited through DM. OR had a high recruitment rate, and DM recruited more participants with a low response rate but higher retention rate. Among recruited participants with data (*n* = 47), smoke-free home rules increased by 50.4 percentage points during the study period (*p* < 0.001). Among recruited participants who had a vehicle (*n* = 38), smoke-free car rules increased by 37.6 percentage points (*p* < 0.01) and comprehensive smoke-free rules rose 40.9 percentage points (*p* < 0.01). Home SHS exposure declined, and within-person increase in smoke-free home rules was significantly related to less home SHS exposure (*p* < 0.05). It is feasible to adapt and implement the evidence-based SFHP intervention through a national cancer program, but the current pilot demonstrated recruitment is a challenge. DM produced a low response rate and therefore OR is the recommended recruitment route. Despite low recruitment rates, we conclude that the SFHP can successfully increase comprehensive smoke-free rules and reduce SHS exposure among socioeconomically disadvantaged households with children recruited through a NBCCEDP.

## 1. Introduction

Exposure to secondhand smoke (SHS) has declined in the U.S. [1], but SHS is common among youth, as nearly 40% of children and one-third of adolescents exhibit biological evidence of recent SHS exposure [2,3]. There are also marked disparities in SHS exposure. Low-socioeconomic status (SES) is associated with higher prevalence of SHS exposure, and SHS exposure is more common among populations of color [4]. Smoke-free policies can prohibit smoking in public places and reduce SHS exposure, and such policies have increased across the U.S. [5]. However, smoke-free public policies do not necessarily apply to *private* spaces, and SHS exposure occurs most commonly in private locations, particularly for children [6,7,8]. The most critical private locations are homes and cars [7,9,10]. Voluntary smoke-free rules in homes can reduce SHS and tobacco use [4,6,7,10]. Yet disparities in smoke-free home rules persist [4,6,11,12]. Low-SES populations are less likely to implement smoke-free home rules [4,7]. Moreover, over 80% of smokers do not restrict smoking in both the home *and* car, and adults living with a child in the home are not more likely to implement smoke-free rules in cars [7,13,14]. Smoke-free rules in both homes and cars, which we define as comprehensive smoke-free rules, are optimal and they are more protective for children compared to smoke-free rules that are less than comprehensive (e.g., prohibiting smoking in the home or car alone) [10,15]. Implementing comprehensive smoke-free rules can help to protect against the harms of SHS exposure and tobacco use relative to no or partial smoke-free rules [10,16], but low-SES smokers and nonsmokers are less likely to implement comprehensive smoke-free rules [7,10]. This study aimed to increase smoke-free rules in homes and cars, focusing on disparities linked to socioeconomic disadvantage.

### Current Study

There is a dearth of research on population-based programs that can successfully increase voluntary comprehensive smoke-free rules among households, and particularly households with children. Scalable evidence-based interventions (EBIs) exist that can increase smoke-free rules in homes [17,18]. However, these intervention materials have focused primarily on smoke-free rules in homes only [12,18,19,20]. Moreover, similar to many EBIs, limited research has tested implementation and dissemination of evidence-based smoke-free interventions in national programs that serve low-income populations and offer potential for population impact. We utilized and adapted the evidenced-based program called the Smoke-Free Homes: Some Things are Better Outside Program (SFHP) that initially focused on increasing smoke-free home rules among 2-1-1 callers (which is a central call center that provides a range of services to low-income households in the U.S.) [18,21]. SFHP consists of direct mail materials and a proactive coaching call that are rooted in health behavior theory. The program recruited participants by using a referral system in collaboration with the 2-1-1 call center (i.e., operators recruit eligible participants who call 2-1-1 for unrelated reasons), and the intervention has increased smoke-free home rules among low-income populations through multiple 2-1-1 call centers [12,21]. Yet more research is needed on how the intervention translates across settings [12], and results for smoke-free cars have not been a central focus [18]. Adapting the program to address smoke-free rules in cars is an important area of research [21]. We adapted the SFHP to address comprehensive smoke-free rules and tailored the materials for households with children. For this pilot project, we implemented the intervention through “Sage”, Minnesota’s National Breast and Cervical Cancer Early Detection Program (NBCCEDP). NBCCEDPs serve un- and underinsured women, and the household income limit for program eligibility is at or below 250% of the U.S. federal poverty level [22,23,24]. Sage provides free breast and cervical cancer screening services to over 10,000 unique women each year [24]. Using a single group over time (no comparison group), we tested how the evidence-based SFHP translated to the NBCCEDP setting by focusing on both implementation and impact results. This pilot project had two main goals: (a) test whether the SFHP could be implemented within Minnesota’s NBCCEDP, focusing on two different recruitment routes, as well as developing new intervention materials that directly address smoke-free rules in homes and cars among socioeconomically disadvantaged households with children, and (b) examine over time how smoke-free rules change using longitudinal data from recruited participants.

## 2. Materials and Methods

### 2.1. Study Context

The intervention was implemented through Sage, which is housed within the Minnesota Department of Health (MDH), from January 2019 through October 2019. Sage has a call center staffed by patient navigators that allows the program to have direct contact with Sage patients through proactive and reactive outreach [25,26].

### 2.2. Participants and Eligibility

**Figure 1:**
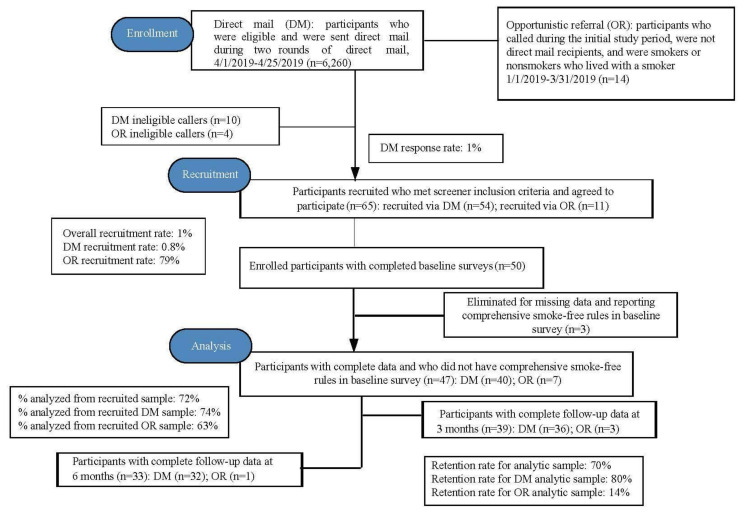
Participant flow chart.

Since NBCCEDPs target low-income females, the current program focused on a sample that was disproportionately female, and the Sage participants were age 18 or older. Patient navigators followed a script based on previous research [12,17,18]. The script allowed patient navigators to confirm specific personal information concerning smoking status, living arrangements, and whether the household had comprehensive smoke-free rules. Patient navigators also determined callers’ decisions about participation and provided details about confidentiality and consent. The flow of participants through the study is presented in Figure 1. Eligible participants for the current study were low-income smokers and nonsmokers who live with a smoker (i.e., based on federal guidelines for NBCCEDPs stated above). Participants were eligible if they lived in a home without comprehensive smoke-free rules; and one person per household was eligible to participate in the study. We only recruited households with children under age 18, and we did not require that the one participant from each household be a parent. We defined a household with children as any household where children lived in the house full-time or visited on a regular basis (e.g., grandchild visiting grandparent). Households with visiting children had more smokers living in their household and fewer people in their personal network that smoked than households where children lived; there were no differences between these two groups in baseline levels of smoke-free rules (and analyses showed no differences in implementation or impact outcomes). Patient navigators in the Sage call center administered baseline surveys at initial time of enrollment to determine eligibility.

### 2.3. Recruitment

We utilized two recruitment routes. First, we developed a direct mail (DM) approach that has not been utilized in previous studies of SFHP. Using data from Sage’s central database, we sent mailers to previous Sage patients who were smokers and nonsmokers who lived with a smoker. We limited the sample to Sage patients using data from July 2015 to February 2019. It was not possible to determine if all Sage patients were eligible for the current pilot project using the Sage database, and therefore, we used available data to create the mailing list based on likelihood of eligibility. Final eligibility was determined by patient navigators after participants called. We developed new mailers that consisted of messages and graphics rooted in the SFHP theme of “Some Things are Better Outside”, and we tailored the messages for households with children. These mailers are presented in supplemental material. Mailers prompted participants to call Sage’s toll-free phone number. Mailers also outlined the incentive that participants would receive for participating in the program (see Supplemental Figure S1), and participants were informed incentives were associated with filling out surveys and not contingent on implementation of smoke-free rules. Participants were informed they could receive up to $30 in three increments (i.e., $10 just for making the first call, and $20 for subsequently filling out follow-up surveys). We incentivized completion of the surveys, which was supported by previous research on the SFHP [19]. Second, we used an opportunistic referral (OR) recruitment method. OR consisted of the Sage call center opportunistically offering the intervention to eligible participants who called about cancer screening services. Sage patient navigators obtained the smoking status of callers and offered the program to self-reported smokers and nonsmokers who live with a smoker. Sage patient navigators used a script from the original SFHP to determine eligibility for participants recruited through OR, which is the recruitment approach that has been utilized in previous studies of the SFHP [18].

### 2.4. Intervention

The Smoke-Free Homes Program (SFHP) consists of three mailings and a coaching call [18]. There were 2-week intervals between intervention components: mailing 1, coaching call, mailing 2, and mailing 3. The theme of the intervention materials is “Some Things Are Better Outside”, and the materials are rooted in multiple health behavior theories that address environmental cues and reinforcement of actions related to creating smoke-free rules [18]. We utilized the original SFHP mailings, but we made adaptations by including new inserts that addressed smoke-free car rules and were tailored to households with children, as well as by generating a coaching e-mail rather than a coaching call. The two new inserts were included in the first and third intervention mailings. The e-mail addressed smoke-free homes and cars, and it was not tailored to the stage of change of the participant. These new inserts are presented in supplemental material (see Supplemental Figures S2 and S3). It is important to also note that all intervention delivery and materials were conducted by consulting the implementation manual, and there was no training provided by SFHP staff since this was a small-scale pilot project. For participants that did not have an e-mail address, coaching letters were sent via mail. In total, each participant received three SFHP mailings, as well as a coaching e-mail or letter.

### 2.5. Data

Data came from reports generated by Sage patient navigators as well as baseline and follow-up surveys conducted in REDCap. For participants who did not have an email address, surveys were sent and returned via mail. There were three surveys in total: baseline, and 3- and 6-month follow-up surveys. All survey questions were generated using verified metrics from the original SFHP materials [12,17,18].

All subjects gave their informed consent for inclusion before they participated in the study. The study was conducted in accordance with the Declaration of Helsinki, and the protocol was approved by the Minnesota Department of Health and University of Minnesota’s Institutional Review Boards.

### 2.6. Outcomes

#### 2.6.1. Implementation Measures

Using the Reach Effectiveness Adoption Implementation Maintenance (RE-AIM) framework for program evaluation to assess implementation and impact outcomes [27], we included measures of program fit (i.e., for adoption in RE-AIM framework) from self-report surveys from Sage patient navigators. For reach, we assessed response and recruitment rates, comparing DM and OR recruitment routes. These are distinct outcomes as response rate corresponds with the response to the DM recruitment method, and recruitment rates correspond to both DM and OR (number of patients recruited relative to potentially eligible participants who were not recruited). We examined predictors of calling for the DM group using a binary outcome of call response (1 = called, 0 = did not call). We also assessed fidelity (implementation in RE-AIM framework) through participant self-report of engagement via a count measure that summed binary responses (1 = yes, 0 = no) to questions regarding whether or not the participant or someone in their home did any of the following in the previous 3 months: came up with a list of reasons for making their home smoke-free; came up with a list of reasons for making their car smoke-free; talked with their family or household members about making their home smoke-free; talked with their family or household members about making their car smoke-free; signed a pledge; posted the pledge; put up provided signs in the home; used provided stickers in the home; and used provided stickers or decals in the car. The measure was summed to capture the total number of intervention components utilized by participants (range = 0 to 9).

#### 2.6.2. Impact Measures

Following previous research [7,18], smoke-free home rules were captured by asking which statement best describes participants’ rules about smoking in their place of residence (not including decks or porches): there are no rules about smoking inside their home; smoking is not allowed anywhere inside their home; smoking is allowed in some places or at some times; smoking is allowed anywhere inside their home. We dichotomized the measure (1 = does not allow smoking anywhere, 0 = other). We asked this question in regards to combustible products, and followed the question with smoke-free rules about vaping products. We did not include the measure about vaping rules because there was no variation (almost all participants reported that they did not allow vaping or reported no one vaped in their household).

Smoke-free car rules were measured by asking participants the following question: “What about smoking in your household vehicles (cars or trucks)? Would you say… There are no rules about smoking in the vehicles; smoking is sometimes allowed in some vehicles; smoking is never allowed in any vehicle; you don’t have a vehicle.” Smoke-free car rules were measured for participants who owned vehicles via a dichotomous measure (1 = smoking is never allowed in any vehicle, 0 = other). Following previous research [7,10], comprehensive smoke-free rules were measured by a dichotomous measure for participants who owned vehicles (1 = smoke-free rules in homes and cars, 0 = other). SHS exposure was a dichotomous metric (1 = yes, 0 = no) based on whether participants reported that there were people smoking in their home within their presence during the previous 7 days.

### 2.7. Covariates

Table 1 shows the covariates included in implementation analyses (i.e., assessing response to DM): race/ethnicity (white, African American, American Indian/Native American, Asian, Hispanic, and other), sex (1 = female, 0 = male), and a measure of individual and household smoking status separated into three dummy variables: smoker living with a nonsmoker, nonsmoker living with a smoker, and smoker living with another smoker. We also included a continuous measure for age (range = 19 to 89). These descriptive statistics in Table 1 are for the sample sent DM.

Table 2 shows the covariates included in impact analyses. We included a binary measure for race/ethnicity (white versus other). For this sample, we used a race/ethnicity measure of white versus other due to the smaller sample size. (A measure for sex was omitted from models due to sample size.) We included a binary measure for whether participants owned their dwelling unit (1 = yes, 0 = other). Marital and cohabitation status (1 = married or cohabiting, 0 = single), education (1 = high school degree or less, 0 = more than high school), and employment at baseline (1 = full- or part-time employment, 0 = not employed) were measured via binary measures. We included a poverty metric based on self-report income and household size using 2018 federal poverty level thresholds set by the Department of Health and Human Services (1 = below federal poverty level, 0 = above federal poverty level). We included a dichotomized measure for whether a household included a child under age 18 full time versus a household where a child visits regularly.

We included a measure of the number of smokers in participants’ personal networks by asking, “Think about your friends and relatives, not just those you might live with. How many of your friends and relatives are smokers? Would you say… all, most, about half, less than half, a few, none.” We used a dichotomized measure to assess participants whose network was half or more smokers versus less than half. We also included a smoking status measure of whether participants were daily smokers at baseline. Since smoking status can change over time, we included smoking status as both an individual-level and a time-varying covariate (see Statistical Analyses). Age was a continuous measure (38 to 68). We included the number of smokers within the household by asking, “Including yourself, how many smokers live in your home?” The range was 1 to 4. These descriptive statistics in Table 2 are for the recruited sample.

### 2.8. Statistical Analyses

Descriptive statistics and logistic regression were used to examine implementation outcomes. First we examined recruitment rates and differences across DM and OR. For impact results (effectiveness in RE-AIM framework), we examined smoke-free rules at 3 time points: baseline, as well as 3 and 6 months after intervention. We used hierarchical linear modelling (HLM) to assess changes in smoke-free rules and SHS exposure over the study period [28]. Since this was a pilot project and we did not have a comparison group, HLM models allow for the examination of the change in the rate of smoke-free rule adoption within the study period, providing a more rigorous significance test that accounts for variance at both time and individual levels and an optimal means for assessing program impact without using an experimental design [28]. We used two-level HLM models that allowed for residual variance for data over time and at the individual level, and they accounted for dependence in error terms by analyzing time and individuals as separate levels of data and by including random effects [28]. A time measure was included in the level-1 equation (time level) in order to capture the growth curve. Research shows that two-level models with a sample size of 30 or more at level 2 provides unbiased and accurate estimates of regression coefficients, variance components, and standard errors (but standard errors at level 2 are at risk of being too small in samples with less than 50 units at level 2) [29]. The models were logistic regression models due to the binary outcome. In order not to saturate HLM models, we ran a series of sequential models to test the growth curve in smoke-free rules and SHS exposure not adjusting and adjusting for covariates (see Supplemental Table S1). Model 1 only included the coefficient for time, while Model 2 included time and baseline demographic variables, and Model 3 included time and smoking-related variables (and all time-varying measures). Finally, we used HLM to examine how within-person change in smoke-free home rules related to SHS exposure during the study period (presented in text). In order to analyze this relationship, we included a time-varying measure of smoke-free home rules at the time level (level 1) that was group-mean centered and a grand-mean centered measure of smoke-free home rules at the individual-level; this allowed for the coefficient at the time level to capture within-person change [28]. We used the same method to control for time-varying smoking status in the primary HLM analyses that examined change in smoke-free rules. Multiple sensitivity analyses were also included.

## 3. Results

### 3.1. Participants

Participant characteristics for those who received DM materials are listed in Table 1. Mean age was 51.7. The sample was 39.4% white, 22.9% American Indian, 21.5% Hispanic, 12.0% African American, and 2.6% other ethnicities/races. Most participants were female (95.5%). Approximately 28.5% of the participants were smokers who lived with a nonsmoker, 18.7% were smokers who lived with another smoker, and the majority were nonsmokers who lived with a smoker (51.8%).

Table 2 shows participant characteristics for those who were successfully recruited and were included in impact result analyses: mean age was 54.9, 39.1% were white, and 60.9% were other ethnicities/races. Most participants were female (95.7%). Most participants rented their living space (36.2% owned their dwelling unit), and there was an even split between married/cohabiting and single participants (43.5% were married/cohabiting). Approximately 49% had a high school education or less, 32.6% were employed at baseline, and the majority had household incomes below the federal poverty level (61.9%). The sample was 52.2% households that had a child living within the household full time (versus having a child visit often), and 68.2% had a personal network that was half smokers or more. Approximately 69.6% of participants were daily smokers at the study’s baseline, and the average number of smokers in their household was 1.64 (range 1–4).

### 3.2. Implementation Results

All Sage staff (100% of staff) agreed that: SFHP was easy to adapt to the Sage population, implementation was not difficult, it would be easy to make the program a permanent part of Sage, SFHP was consistent with Sage’s mission, they were comfortable recruiting and conducting eligibility screening, tasks for the intervention were similar to normal Sage-related tasks, their skills were a good fit for SFHP, it was not time-consuming to deliver SFHP, and SFHP was seen as an important part of Sage’s mission by leadership. Overall, 75% of staff enjoyed working on the program and felt a personal responsibility for SFHP’s success.

In terms of DM response rate, reach corresponded to previous research on DM response rates (a total response rate of 1%). A total of 64 eligible participants were recruited, and the number of participants at each intervention stage is reported in Figure 1. Of the 6274 participants contacted via DM or OR, 65 were recruited (overall recruitment rate of 1%). Approximately 83% of participants were recruited through DM, with 54 participants recruited out of the 64 who responded to the DM materials (DM recruitment rate based on all 6260 participants sent DM materials was 0.8%, but not all DM recipients were potentially eligible, as full eligibility was determined when respondents called the Sage call center; see Figure 1). OR had a high recruitment rate at 79%, which was based on the number of participants who called the Sage call center for other reasons and were opportunistically offered the program if they were eligible. OR had a higher recruitment rate, and DM recruited more participants with higher retention (see Figure 1). In terms of predictors of recruitment shown in Table 3, the main predictor of calling in response to DM was personal and household smoking status; no other covariates were statistically significant. There were no other differences between those who responded to DM and those who did not beyond smoking status. Relative to smokers who live with a nonsmoker, nonsmokers who live with a smoker, and smokers who live with a fellow smoker had lower odds of responding (respectively, adjusted odds ratio [AOR] = 0.04, 95% CI = 0.01, 0.32; and AOR = 0.25, 95% CI = 0.09, 0.71), adjusting for covariates. In terms of participant engagement, the average number of intervention components executed by participants at follow-up 1 was 4.53 (SD = 2.56; range = 0–9), and the average at follow-up 2 was 4.63 (SD = 2.63; range = 0–9).

### 3.3. Impact Results

The prevalence of smoke-free rules in homes went from 12.8% at baseline to 69.2% at 3-month follow-up and 63.2% at 6-month follow-up. As shown in Table 4, HLM models with only the time trend showed that the rate of increase in smoke-free homes within the study period was nonlinear and statistically significant (*p* < 0.001). This increase in smoke-free home rules was statistically significant adjusting for covariates in models presented in Supplemental Table S1. Among participants who had a vehicle, the percent of individuals with smoke-free car rules went from 7.9% at baseline to 38.5% at 3-month follow-up and 45.5% at 6-month follow-up. The rate of increase in smoke-free car rules within the study period was statistically significant (*p* < 0.05), adjusting for covariates in separate models presented in Supplemental Table S1. However, the relationship had a *p*-value of 0.06 in Model 3 in Supplemental Table S1. Finally, comprehensive smoke-free rules increased from 0% at baseline to 30.8% at 3-month follow-up and 40.9% at 6-month follow-up. Significance tests for this time trend from HLM models showed that this trend was statistically significant (*p* < 0.01), even adjusting for covariates in separate models presented in Supplemental Table S1.

Results for SHS exposure are shown in Table 4. The percent of participants who reported exposure to SHS from someone within their home in the past week declined from 78.3% at baseline to 32.5% at 3-month follow-up and 37.5% at 6-month follow-up. HLM showed that this time trend was nonlinear, and that the trend was statistically significant (*p* < 0.01), even after adjusting for covariates presented in Supplemental Table S1. Change in smoke-free home rules during the study period was significantly related to a decline in SHS exposure, accounting for individual-level differences in smoke-free home rules (log-odds for relationship between smoke-free home rules and SHS exposure = −1.69, SE = 0.71, *p* = 0.02), and multivariable analyses showed this relationship remained statistically significant adjusting for covariates.

Sensitivity analyses examined whether any HLM results changed after including a measure for DM versus OR; no results changed after including these additional covariates, and recruitment method was not significantly related to smoke-free rules over the study period. Sensitivity analyses also included intent-to-treat analyses that coded all missing values as no home, car, or comprehensive smoke-free rules for participants who were lost due to attrition at follow-ups. Results remained similar for all three smoke-free rules outcomes: home rules increased and then decreased (log-odds for linear term = 5.34, S.E. = 1.33, *p* < 0.001; log-odds for squared term = −2.05, S.E. = 0.57, *p* < 0.001), car rules increased (log-odds = 0.80, S.E. = 0.38, *p* < 0.05), and comprehensive rules increased (log-odds = 1.41, S.E. = 0.54, *p* < 0.01).

## 4. Discussion

Using the RE-AIM framework, we found that adoption, implementation, and effectiveness outcomes were encouraging for the current pilot project. One of the main purposes of the current pilot was to tailor the SFHP to Minnesota’s NBCCEDP. We were able to successfully train NBCCEDP staff to screen for eligibility among potential participants, as well as to recruit eligible participants. Yet it is important to note that recruitment was challenging, which potentially stemmed from a few aspects of the current pilot project (see Limitations section below); and consequently, reach results from the current pilot were a weakness. Responses from program staff and adaptation results demonstrated that the adapted version of the SFHP program was feasibly implemented (e.g., successful buy-in and adoption) within a national program that serves low-SES populations; consequently, more rigorous randomized trials should test this adapted version of the SFHP in other NBCCEDPs across the U.S. Another primary aim was to develop new materials, and we successfully developed materials that addressed smoke-free rules in both homes and cars, focusing specifically on households with children. We eliminated the coaching call that was associated with the original SFHP, and we did not utilize training from the intervention designers, which demonstrates the SFHP could feasibly be disseminated without these intervention components and training, depending on a site’s budget and capacity. We also developed new inserts for the mailed SFHP intervention that promoted smoke-free car rules. Participants who received this adapted version of the SFHP intervention reported marked increases in the rate of adoption of smoke-free rules in homes, cars, and comprehensive smoke-free rules during the study period.

We developed DM recruitment materials, which used themes from the original SFHP materials as well as new themes that included comprehensive smoke-free rules with a focus on their benefit for children. We conclude that DM led to less than ideal reach results for the current pilot. We found most participants were recruited through DM, and the response rate of 1% to the DM mailing corresponded to previous DM research conducted through Minnesota’s NBCCEDP. We expected a response rate for DM not to be higher than 2% as most DM campaigns, including those implemented within the Sage population, have produced response rates between 1% and 2% [23,24,26,30,31]. The DM route associated with this SFHP pilot is capable of achieving the lowest expected response rate. Since the opportunistic referral route has been an effective recruitment method in previous studies of the Smoke-Free Homes Program, one of the central tests of the current pilot was to introduce an additional recruitment method—direct mail—in order to gauge whether it is a viable additional option. DM is relatively cheap, and with a large population, a response rate of 1% to 2% typically can still provide a sizable number of participants if a mailing list is accurate. For instance, a 1% response rate associated with a national scale up within NBCCEDPs across the country would potentially lead to thousands of households implementing smoke-free rules and reducing SHS for thousands of children.

Yet, our conclusion is that the OR recruitment is more reliable. The OR recruitment route had a high recruitment rate, and this route has been used in previous studies that established the efficacy of the SFHP. It has already been established that OR is an effective route for recruiting participants to the SFHP [12,17,18]. While DM recruited more participants with higher retention, which corroborates previous research on tobacco cessation projects implemented through Minnesota’s NBCCEDP [24], we conclude that OR offers access to more potential participants and this route should be used in future efforts to implement the SFHP. We also contend that OR should be used for a longer period of time relative to the current pilot, and ideally, NBCCDEPs could incorporate the SFHP into daily operations, which would allow for extended periods of recruitment. During a two-year period, Minnesota’s NBCCEDP used the OR recruitment route to recruit 4550 low-income smokers out of approximately 23,000 callers that called the Sage call center for a smoking cessation intervention, and this Sage study found that OR recruited a substantially larger number of participants relative to DM [24]. A central aim of the current pilot was to test how well DM would reach individuals at one time, and while the current pilot demonstrated the feasibility of implementing DM, we concluded that DM is not the ideal recruitment for the SFHP route compared to OR.

### 4.1. Limitations

As noted, the main limitation of the current pilot project was reach. The intervention did not recruit a substantial number of patients. Aspects of the current pilot project that could have influenced this recruitment rate. First, the entire study period spanned 9 months; however, recruitment occurred over a much shorter time period. With a larger budget and timeframe, the current pilot project could be expanded to include a longer recruitment period, particularly for the OR recruitment route. Only 14 eligible Sage callers were offered the SFHP during the timeframe for the current pilot project through the OR route. This was the result of a small number of eligible participants calling the call center for other services during the OR recruitment period, which spanned approximately 3 months.

In terms of DM recruitment, DM was more expansive than OR for the current pilot project, but the data from the Sage database that was used for the DM mailing list did not allow us to narrow the scope of the mailing list or determine eligibility beyond smoking status, number of people in household, and whether participants lived with a smoker. Multiple participants who received the DM materials were potentially not eligible for the current pilot intervention. Response and recruitment rates for DM could be higher with a more targeted mailing list that uses more eligibility criteria. Alternatively, recruitment could improve in future implementation if programs have larger mailing lists. Previous research on the SFHP shows that recruitment can be difficult in situations without access to large numbers since eligibility criteria are more specific for the SFHP relative to other programs (e.g., programs devoted strictly to smoking cessation). A key finding from the current pilot study highlights this issue: the most prevalent group that received DM were nonsmokers who lived with a smoker (over 50% of the mailing list), but the group most likely to call in response to DM was relatively small—i.e., smokers who live with a nonsmoker. Moreover, we limited our sample to include only households that had children living within the household full time or households that had children visit regularly. Future efforts to implement the SFHP through programs such as NBCCEDPs should follow previous research on the SFHP that includes all households regardless of whether children are present.

Future studies should also consider other methods that may increase recruitment; for instance, previous research conducted through Sage shows that using DM with a proactive call from Sage patient navigators (i.e., patient navigators use phone numbers from the Sage database to call patients who do not immediately respond to DM) can increase recruitment rates [26]. Another limitation of the current pilot was that due to the timeframe and pilot budget, we used a pre-post study design and subsequently there was no control group. Future studies should expand on the current pilot and utilize a randomized control trial design. For this particular pilot study, we aimed to recruit participants who were motived to implement smoke-free rules, and we found no differences between OR and DM in terms of the outcomes, but these two recruitment routes have been shown to recruit different participants according to motivation for behavioral change [24]. Results should be interpreted in this vein—i.e., participants were not randomized to a treatment and control condition, and therefore, common problems associated with the generalizability of results from public health program evaluations should not be ignored (e.g., selection effects, response biases, etc.). Our implementation results are only indicative of responses for the population that was examined in the current pilot, and they do not represent results that may occur in more rigorous tests of SFHP implementation. Additionally, our impact results do not reflect true treatment effects representative of the NBCCEDP population. Our within-person change results reflect only the potential impact of the intervention for this particular pilot and the subpopulation of NBCCEDP patients that were recruited. All implementation and impact results therefore should be considered as preliminary, and future randomized trials should be conducted to generate true experimental effect sizes that eliminate biases such as selection effects.

### 4.2. Implications for Public Health Practice

While utilizing the NBCCDEP context could potentially work for reaching low-income households throughout the U.S., other delivery sites should also be considered in order to increase reach of the SFHP. Future implementation of the program would ideally be integrated into the ongoing program of NBCCEDPs and would not require surveys, and therefore, incentives for filling out surveys would not be required. Yet, it is important to note that other dissemination trials of the SFHP showed that the program was cost-effective even with incentives included in the cost analysis [19]. Similar to other smoking-related objectives in the NBCCEPDs (e.g., referring smokers to the state quitline), the SFHP could fit within the ongoing programmatic work of NBCCEDPs and staff can buy into the program due to its goals. Yet, other options should also be pursued for national scale up efforts, such as delivery within primary care clinics. Moreover, partnering with other tobacco-related programs, such as state free quitlines, or programs that serve socioeconomically disadvantaged populations, such as state Medicaid programs, could also be fruitful avenues. Medicaid data would allow the use of direct mail and OR recruitment methods within a larger population [23]. The Medicaid program serves a population with higher smoking prevalence compared to the general population [32], and it disproportionately serves low-income households with children, and particularly children who have a potentially higher prevalence of SHS exposure [33].

## 5. Conclusions

Comprehensive smoke-free rules (smoke-free rules in homes and cars) are optimal, particularly for children [10]. In the current pilot project, we utilized and adapted the evidenced-based SFHP [18] and implemented the intervention through Sage, Minnesota’s NBCCEDP. We successfully tailored the intervention to the Sage program and developed new materials to address smoke-free rules in both homes and cars, focusing specifically on households with children. We utilized two recruitment routes, including OR, which had been used in previous studies of SFHP, as well as the DM recruitment route. We recommend use of OR recruitment based on the recruitment rate found in the current pilot. The intervention was effective as it increased comprehensive smoke-free rules, as well as reduced SHS exposure in the home. Future research should test ways to disseminate across programs and increase recruitment numbers.

## Figures and Tables

**Table 1:** Demographic Details of Direct Mail Recipients.

	% (n)		
Race/ethnicity			
White	39.41%	(2375)	
African American	11.98%	(722)	
American Indian/Native American	22.86%	(1378)	
Asian	1.68%	(101)	
Hispanic	21.50%	(1296)	
Other	2.57%	(155)	
Female	95.53%	(6011)	
Personal and household smoking status			
Smoker lives w/nonsmoker	28.51%	(1857)	
Nonsmoker lives w/smoker	51.81%	(3260)	
Smoker lives w/smoker	18.67%	(1175)	
	**Mean (SD)**		**Range**
Age (in years)	51.70	(11.61)	(19–89)

*Notes*: N = 6292; sample size varies by variable based on missing data.

**Table 2:** Descriptive Statistics for Analytic Sample with Full Baseline Data.

	% (n)		
Demographics			
Race/ethnicity			
White	39.13%	(18)	
Other	60.87%	(28)	
Female	95.65%	(44)	
Own home (vs. rent)	36.17%	(17)	
Married or cohabiting (vs. single)	43.48%	(20)	
Education (≤high school vs. >high school)	48.89%	(22)	
Employed at baseline	32.61%	(15)	
Below federal poverty level ^a^	61.86%	(24)	
Child lives in house full time (vs. other)	52.17%	(24)	
Personal network is half or more smokers	68.18%	(24)	
Daily smoker at baseline	69.57%	(32)	
	Mean (SD)		Range
Age (in years)	54.85	(6.87)	(38–68)
Number of smokers in household	1.64	(0.72)	(1–4)

*Notes*: N = 47; sample size varies by variable based on missing data; ^a^ Based on income and household size, and national guidelines set by the Department of Health and Human Services for 2018.

**Table 3:** Logistic Regressions Predicting Call Response for Direct Mail Recipients.

Variables		Odds Ratio	95% CI
Race/ethnicity			
	White (vs. other)	0.678	(0.384, 1.197)
Personal and household smoking status			
	Smoker lives w/nonsmoker (reference)		
	Nonsmoker lives w/smoker	**0.043**	(0.006, 0.315)
	Smoker lives w/smoker	**0.253**	(0.090, 0.707)
Age (in years)		1.028	(0.999, 1.057)

Notes: Bolded odds ratios are statistically significant (*p* < 0.01). N = 5353. A total of 939 individuals were missing data on race/ethnicity; results did not change when race/ethnicity was removed from the model, except the relationship for age became significant (*p* < 0.05).

**Table 4:** Change in Smoke-free Rules and Secondhand Smoke Exposure Outcomes over Study Period

Outcomes	Proportion with Smoke-Free Rules			Unadjusted Log-Odds for Time Trend
	Baseline	3-Month Follow-Up	6-Month Follow-Up	
Smoke-free rules				
Home ^a^	12.77%	69.23%	63.23%	6.79 ***
				(1.75)
				−2.37 **
				(0.73)
Car	7.89%	38.46%	45.45%	1.22 **
				(0.46)
Comprehensive (home and car)	0.00%	30.77%	40.91%	1.78 **
				(0.65)
Smoke exposure				
Exposed to someone smoking in past week	78.26%	32.50%	37.50%	−7.85 **
				(2.58)
				2.67 **
				(0.99)

Notes. Standard errors are in parentheses; full information maximum likelihood was used with all available data. For home rules, baseline N = 47; For car and comprehensive rules, baseline N = 38; For secondhand smoke exposure, baseline N = 46; ^a^ The time trend for smoke-free home rules was nonlinear and required a polynomial to accurately capture the time trend. *** = *p*-value < 0.001; ** = *p*-value < 0.01. Log-odds, standard errors, and *p*-values are from hierarchical logistic regression models.

## Supplementary Materials

The following are available online at https://www.mdpi.com/1660-4601/17/18/6787/s1, Figure S1: Direct Mail Example, Figure S2: New Insert #1, Figure S3: New Insert #2, Table S1: Change in Smoke-free Rules and Secondhand Smoke Exposure Outcomes over Study Period: Unadjusted and Adjusted Growth Curve Models.
