# Supplementary material for: Reducing Socioeconomic Disparities in Comprehensive Smoke-Free Rules among Households with Children: A Pilot Intervention Implemented through a National Cancer Program
Source: Int J Environ Res Public Health. 2020 Sep 17;17(18):6787. doi: 10.3390/ijerph17186787 (PMC7559315; doi:10.3390/ijerph17186787)
Supplement: Supplementary file 1 [file ijerph-17-06787-s001.zip › Supp Zip/Supp Figure S1, mp.pdf]

DEPARTMENT OF HEALTH

SAGE
MINNESOTA'S CANCER SCREENING PROGRAM

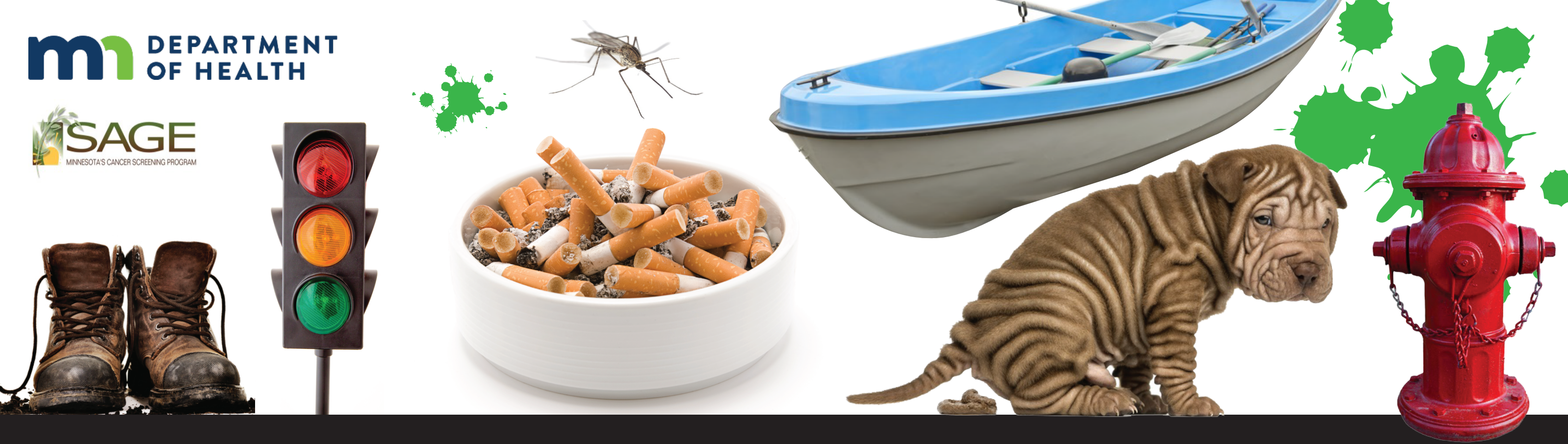

Some things are better OUTSIDE

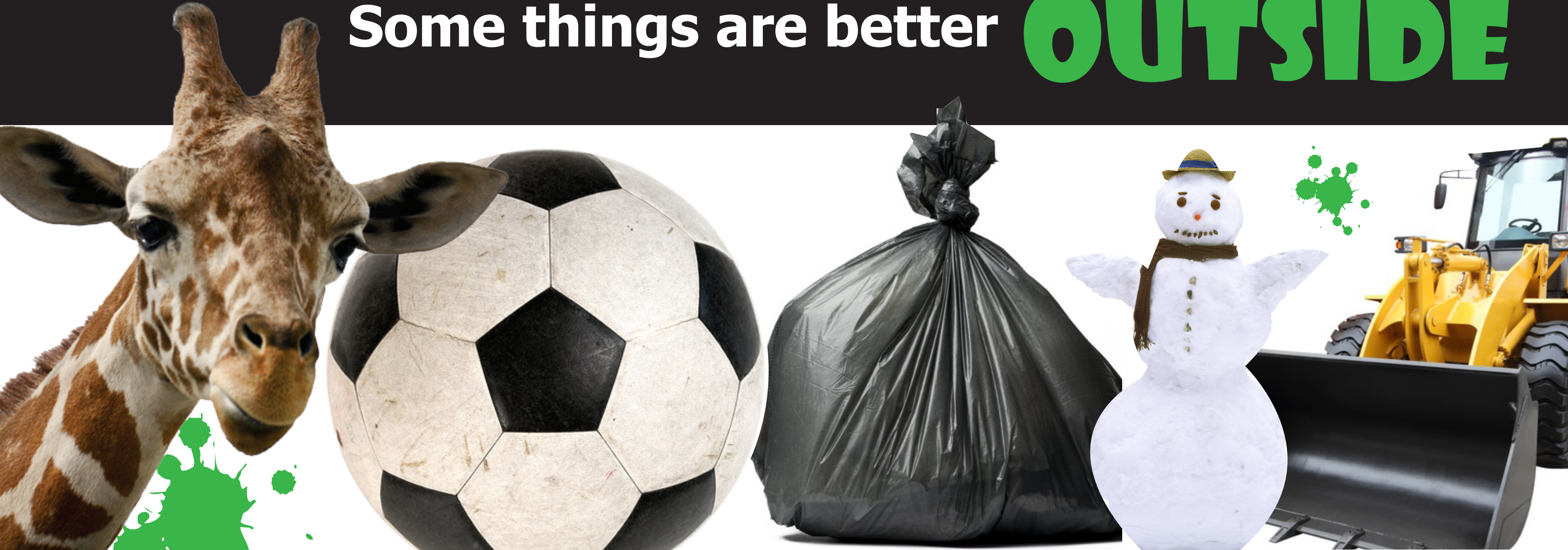

Make your home
HEALTHIER
FOR EVERYONE
(especially the kids)

Keep the SMOKE outside

WHY?

A smoke-free home is good for everyone:

- Keeps your kids from getting sick & missing school
- Reduces your families' risk of getting cancer
- Helps a smoker to cut back or quit
- Makes your home fresher and cleaner

WHO?

Families agree smoke-free homes and cars are better, but still over 1/3 of US kids are exposed to smoke each day.

Bottom line: there is no safe level of second-hand smoke. It belongs outside.

WHAT?

If you are a smoker or live with one (and have kids at home) a new program, **SAGE**, offers resources you can use to take it outside.

The program includes a free toolkit with step-by-step guide on how to maintain smoke-free homes and cars, tips, postcards, and more!

HOW?

Take the first step!

Call **SAGE** now to get started. Mention code "ASDF" or just ask about the smoke-free home program.

1-888-643-2584

GET $10 JUST FOR SIGNING UP
$20 FOR TRYING IT & TAKING OUR SURVEY!
