# Supplementary material for: Reducing Socioeconomic Disparities in Comprehensive Smoke-Free Rules among Households with Children: A Pilot Intervention Implemented through a National Cancer Program
Source: Int J Environ Res Public Health. 2020 Sep 17;17(18):6787. doi: 10.3390/ijerph17186787 (PMC7559315; doi:10.3390/ijerph17186787)
Supplement: Supplementary file 1 [file ijerph-17-06787-s001.zip › Supp Zip/Supp Figure S2, mp.pdf]

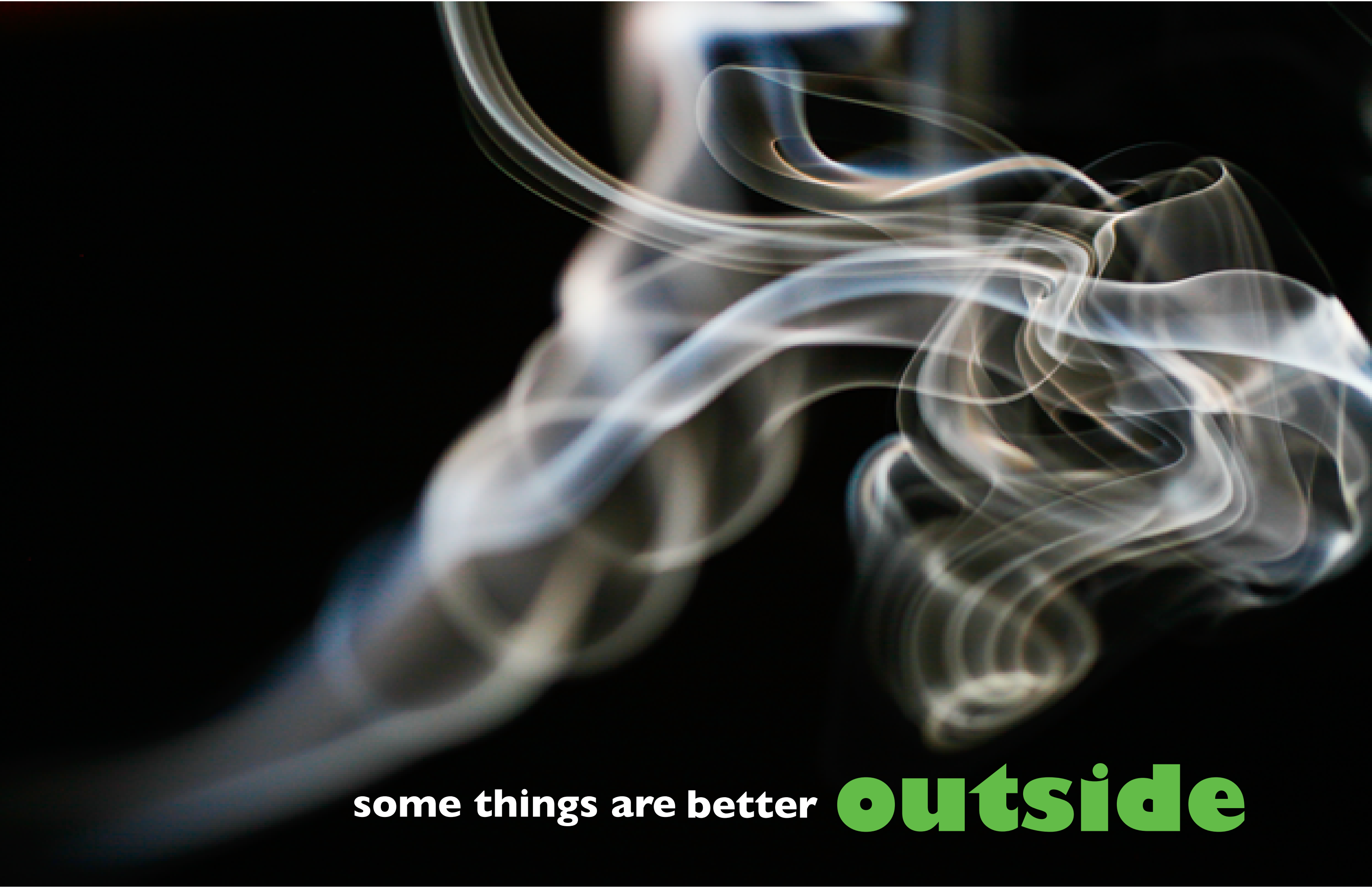
some things are better outside

Make your car HEALTHIER by...

taking it outside

Ways to help make your car smoke-free:

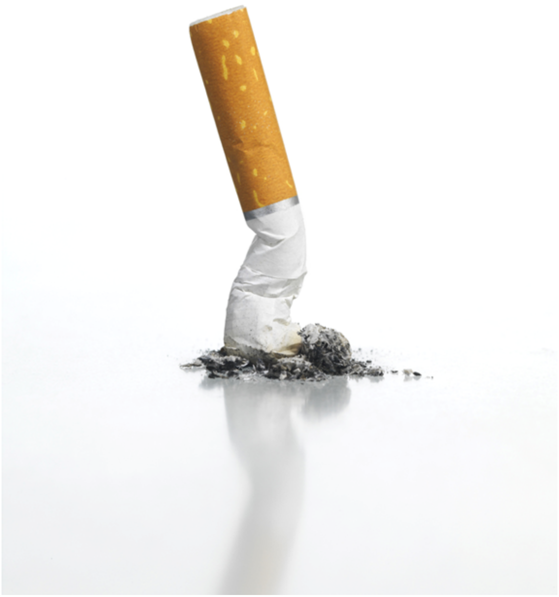

1. Put a Smoke-Free Homes decal on your car window

2. Place Smoke-Free Homes stickers on the inside or outside of your car
