# Supplementary material for: Reducing Socioeconomic Disparities in Comprehensive Smoke-Free Rules among Households with Children: A Pilot Intervention Implemented through a National Cancer Program
Source: Int J Environ Res Public Health. 2020 Sep 17;17(18):6787. doi: 10.3390/ijerph17186787 (PMC7559315; doi:10.3390/ijerph17186787)
Supplement: Supplementary file 1 [file ijerph-17-06787-s001.zip › Supp Zip/Supp Figure S3, mp.pdf]

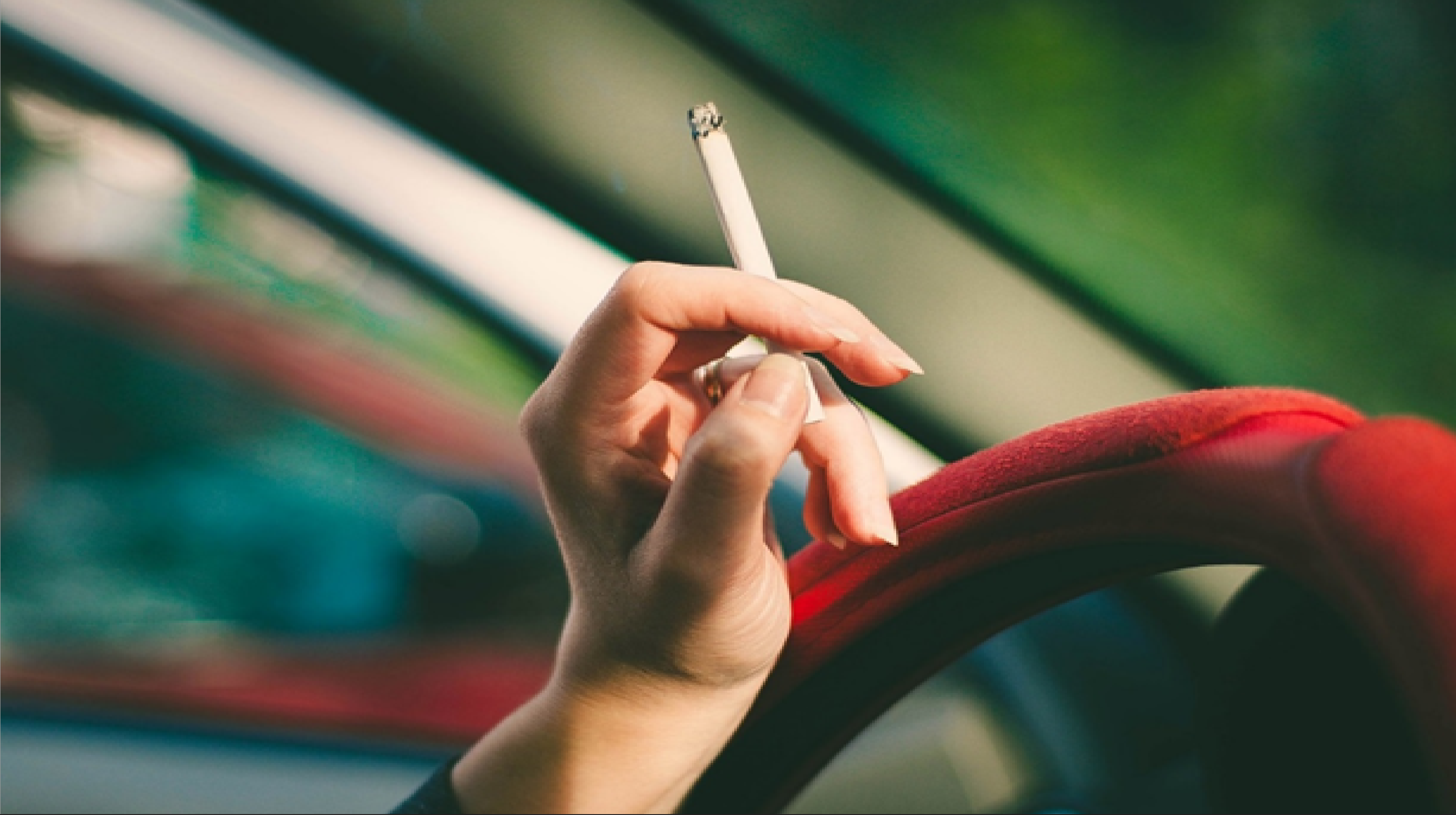

some things are better **outside**

Make your car **HEALTHIER** for everyone (especially the kids) by...

taking it outside

A few minutes in a smoky car = 3 hours in a smoky bar... even with the windows down.

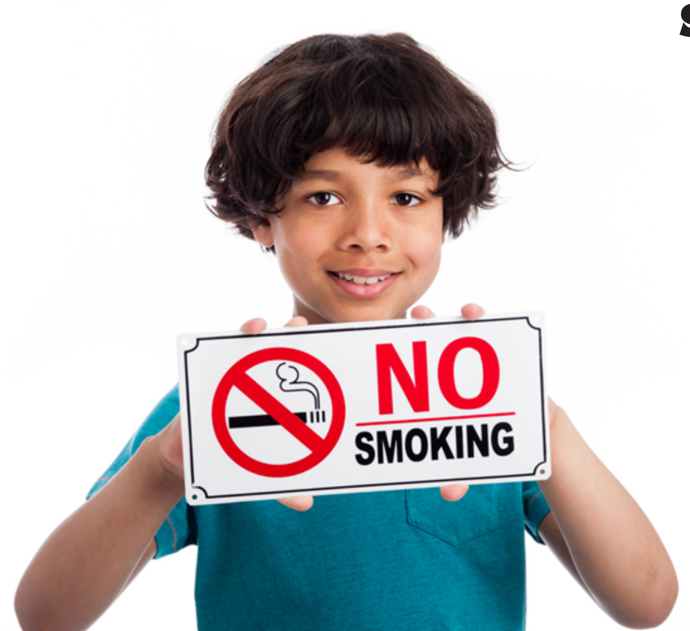

Secondhand smoke in cars:

- No safe level of secondhand smoke in cars
- It causes cancer
- Over one third of US kids are exposed to smoke each day in homes and cars.
